# Beyond the exponential horn: a bush-cricket with ear canals which function as coupled resonators

**DOI:** 10.1098/rsos.220532

**Published:** 2022-10-12

**Authors:** Emine Celiker, Charlie Woodrow, Aurora Y. Rocha-Sánchez, Benedict D. Chivers, Ludivina Barrientos-Lozano, Fernando Montealegre-Z

**Affiliations:** ^1^ University of Lincoln, School of Life and Environmental Sciences, Joseph Banks Laboratories, Green Lane, Lincoln LN6 7DL, UK; ^2^ Tecnológico Nacional de México-I. T. de Ciudad Victoria, Blvd. Emilio Portes Gil No. 1301, Ciudad Victoria, C.P. 87010 Tamaulipas, México

**Keywords:** bush-cricket ear, acoustic trachea, finite-element analysis, laser Doppler vibrometry

## Abstract

Bush-crickets have dual-input, tympanal ears located in the tibia of their forelegs. The sound will first of all reach the external sides of the tympana, before arriving at the internal sides through the bush-cricket's ear canal, the acoustic trachea (AT), with a phase lapse and pressure gain. It has been shown that for many bush-crickets, the AT has an exponential horn-shaped morphology and function, producing a significant pressure gain above a certain cut-off frequency. However, the underlying mechanism of different AT designs remains elusive. In this study, we demonstrate that the AT of the duetting Phaneropterinae bush-cricket *Pterodichopetala cieloi* function as coupled resonators, producing sound pressure gains at the sex-specific conspecific calling song frequency, and attenuating the remainder—a functioning mechanism significantly different from an exponential horn. Furthermore, it is demonstrated that despite the sexual dimorphism between the *P. cieloi* AT, both male and female AT have a similar biophysical mechanism. The analysis was carried out using an interdisciplinary approach, where micro-computed tomography was used for the morphological properties of the *P. cieloi* AT, and a finite-element analysis was applied on the precise tracheal geometry to further justify the experimental results and to go beyond experimental limitations.

## 1. Introduction

Tettigoniidae, also known as bush-crickets, have sophisticated and ultrasound-sensitive ears located in the tibia of their forelegs. Each of their ears is endowed with two tympanal membranes (TMs), which receive the sound on both sides [1]: the external side directly and the internal side through a tracheal tube (the acoustic trachea, AT), which runs along the foreleg. It is widely accepted that the AT is the main input of sound for the majority of bush-cricket species [2–4]. Hence, discerning the tracheal acoustical properties becomes crucial in understanding the workings of the bush-cricket auditory system.

The AT is an air-filled tube that is derived from the respiratory system of the bush-cricket. The main function of this tube is to act as a sound guide, delivering the sound wave which enters it through an opening on the prothorax (the acoustic spiracle) to the TMs. The trachea extends from the thorax to the femorotibial joint (the knee), at which point it enters the tibia and divides into two branches: an anterior branch leading to the anterior tympanal membrane (ATM) and a posterior branch connected with the posterior tympanal membrane (PTM) [5,6]. The mechanoreceptors of the bush-cricket ear (the *crista acustica*, CA) lie on the dorsal wall of the anterior branch [7] and are contained in a haemolymph-based fluid channel (HC), located between the two tympana. In some species, this channel has become modified into a distinct fluid-filled cavity (the auditory vesicle, AV), and the formed AV and CA are also likened to the mammalian inner ear, providing an analogy between the auditory mechanisms of the mammalian and bush-cricket ears [8]. Finally, the tracheal divisions rejoin below the TM and the narrowing AT ends right beneath the ear [9].

Among the known approximately 8100 different bush-cricket species, various differences have been noted in their spiracle size, tracheal design and tracheal length [10]. Nevertheless, the AT generally has an expanded part near the spiracle (the bulla), which either (i) forms a distinct chamber that is attached to the (almost uniform) remainder of the tube, or (ii) is smoothly connected to the trachea, giving the AT a flared horn-shaped geometry [5]. This variation in design has been observed to lead to a deviation between the sound transmission properties of the ATs. The flared horn-shaped geometry, for instance, has been shown to act as a finite exponential horn [6,11,12], since after a certain cut-off frequency, a considerable pressure gain was measured at the distal end of the tube. Whereas for the species having the bulla as a distinct chamber, the AT has been observed to behave as a sharply tuned resonator [4,13]. In [4], it was claimed that the resonant frequency of these ATs is dependent on the length of the tube. Even though an extensive investigation of the biomechanics behind a horn-shaped AT exists in the literature, there are only a limited number of studies related to the workings of the ‘resonator’ AT. Hence, the driving force behind the observed resonance remains largely elusive and certainly merits further investigation. Nevertheless, such an investigation becomes very challenging experimentally, as the small dimensions of the AT do not allow for the non-invasive measurement of the sound pressure distribution inside the tube. Thus, a numerical approach is required for overcoming such experimental limitations.

In addition to sound pressure gain, the bush-cricket AT has also been noted to contribute to a reduction of sound speed [14]. For instance, compared with free-field sound speed in air, the speed of sound was measured to be up to 25% slower after travelling through the tracheal tube of the species *Copiphora gorgonensis* [1], which are endowed with an exponential horn-shaped trachea. As a result, the sound reaches the internal side of the TM with a higher magnitude and delay compared with the external side, leading to the TM acting as a pressure-difference receiver [15]. It is deemed that this pressure-difference receiving system also aids the directional hearing of the bush-cricket [16]. However, to our knowledge, the amount of speed reduction has not been quantified for the species with a ‘resonator’ AT geometry.

Bush-cricket ears are typically adapted to be most sensitive to sounds that are significant for their fitness and/or survival. As is well known, the male will produce an acoustic signal to attract a mate. Accordingly, the bush-cricket AT has been shown to be designed to produce the highest transmission gain at the calling song frequency of a species, allowing the female to hear potential mates more clearly and the male to detect competing conspecific males [5]. In the sub-family Phaneropterinae, which represents about 35% of the species described, the females will also produce calls to answer calling males for mating, leading to a duet between the pair [17]. However, for the majority (approx. 65%) of the species, the females do not respond to the male with a calling song.

For about one-third of the species (2800 of 8100 species) where both males and females call in duet, differences are expected to occur in the frequency sensitivity requirements between the sexes [18]. In this case, it is predicted that the hearing system of each sex will differ, leading to a sexual dimorphism in their AT design [5,19]. Such a difference between the sexes was observed by Stumpner and Heller in [20], where the authors showed that the males of the species *Poecilimon gracilis* are more sensitive in the frequency range 40–50 kHz compared with females. It is argued that this is because the females respond to males in a very narrow time window, and since high frequencies give a better indication of distance, this higher sensitivity allows the males to locate them more effectively. Nonetheless, this difference can easily be attributed to other ecological factors, such as the male bush-crickets being at a higher risk from predators like bats than the females [21]. Hence, further investigations are required to obtain more conclusive evidence related to any differences in the auditory systems of duetting species.

**Figure 1.**
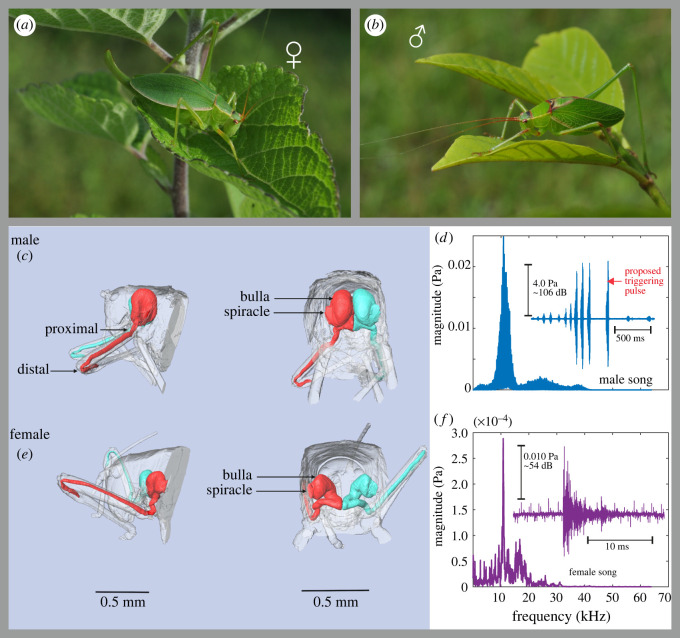
Sexual dimorphism and call description in males and females of *Pterodichopetala cieloi*. (*a*) A female and (*b*) a male bush-cricket *P. cieloi*. The *μ*-CT images of the acoustic trachea morphology of (*c*) a male and (*e*) a female *P. cieloi*, located inside the body of the specimen. The *P. cieloi* (*d*) male and (*f*) female calling songs and their spectral decomposition.

Such a duetting bush-cricket species is *Pterodichopetala cieloi*, which is endemic to northeastern Mexico [22]. Figure 1 demonstrates the dimorphic geometry of the male and female *P. cieloi* AT, where for both of the sexes, the bulla is a distinct chamber (see §3.2 for more details). However, a spectral analysis of the male and female calling song recordings revealed that both have a dominant frequency of 11 kHz (figure 1*d*,*f*).

Using *P. cieloi* as our model system, in this study we test two hypotheses: (i) Despite the dimorphism in the male and female *P. cieloi* tracheae, both the ATs will produce the maximum transmission gain at 11 kHz, the shared calling song frequency; and (ii) The resonant frequency of the AT will be dependent on the coupled transmission properties of the two parts making up the AT, namely the bulla and the tubular part. This investigation was carried out using an interdisciplinary approach. In particular, experimental data were obtained through laser Doppler vibrometer (LDV) measurements of tympanal displacements, which was supported by a finite-element analysis (FEA) conducted on the precise tracheal geometry. With the validation of the numerical results through comparisons with experimental data, FEA was also used to go beyond experimental limitations. The precise tracheal geometry of *P. cieloi* was obtained through micro-computed tomography (*μ*-CT) and has also been used to quantify the morphological differences between the sexes. Further, through numerical results we calculated the amount of sound speed reduction in the *P. cieloi* AT. Both experimental and numerical results were in support of our hypotheses.

## 2. Material and methods

### 2.1. Specimens

In this study, seven male and six female specimens of the bush-cricket *P. cieloi*, endemic to northeastern Mexico, were used for LDV experiments (see §2.5). They were kept in cages at 22–27°C, with a light/dark cycle of 11/13 h, and 70% relative humidity. During captivity, the specimens were fed a mix of pollen, apple, lettuce and water. The specimens generally survived the experiments. Following their natural death, the specimens’ bodies were preserved in 2 ml of Bouin’s solution and then used for three-dimensional *μ*-CT scanning, as described in §2.3.

### 2.2. Calling song recordings

Recordings were made in a sound-attenuated booth (IAC Acoustics, Series 120a, internal length 2.40 m, width 1.8 m and height 1.98 m) at the School of Life and Environmental Sciences, University of Lincoln, at a temperature of 24°C and relative humidity of 60%. Male specimens were placed in a metallic mesh cage (5 cm diameter × 10 cm height) at 10 cm from a 1/8 condenser microphone (G.R.A.S. 40 DP; G.R.A.S. Sound & Vibration, Denmark), connected to a 1/4 Preamplifier (G.R.A.S. 26TC). The microphone was calibrated at 94 dB SPL (re 20 Pa), using a Brüel and Kjær sound-level calibrator (Type 4231, Brüel and Kjær, Naerum, Denmark). Females were recorded in the same way, but males were moved away from the microphone while still being kept in the same acoustic booth as females, so that male acoustic activity would stimulate female singing. Data were obtained via an acquisition board (PCI-6110, National Instruments, Austin, TX, USA) and stored on a computer hard disk at a sampling rate of 512 k-samples/s using the Polytec acquisition software (PSV 9.0.2, Polytec GmbH, Waldbronn, Germany). Sound was analysed using Matlab (R2019a, The MathWorks, Inc., Natick, MA, USA).

### 2.3. Three-dimensional segmentation and morphological measurements

To produce the precise three-dimensional geometry of the AT for the mathematical models and for morphological measurements, six AT of *P. cieloi* (three male and three female) were scanned using a SkyScan 1172 X-ray *μ*-CT scanner (Bruker Corporation, Billerica, MA, USA) with a resolution of 12.9 μm (50 kV source voltage, 200 μA source current, 200 ms exposure and 0.2° rotation steps). For obtaining a series of orthogonal slices, the *μ*-CT projection images were reconstructed with NRecon (v. 1.6.9.18, Bruker Corporation, Billerica, MA, USA).

The three-dimensional segmentation of the ATs were performed with the software Amira-Aviso 6.7 (Thermo Fisher Scientific, Waltham, MA, USA) and exported as stereolithography (STL) files for numerical modelling using established protocols [12,14]. The segmented images were also used for obtaining the AT cross-sectional radius, length and volume measurements through the Center Line Tree module in AMIRA. For the two-dimensional measurement of spiracle size, an Alicona InfiniteFocus microscope (G5, Bruker Alicona Imaging, Graz, Austria) at ×5 objective magnification was used, with a resolution of about 100 nm.

### 2.4. Mathematical model and numerical simulations

The numerical calculations were carried out using the Acoustics module of COMSOL Multiphysics, v. 5.6 [23]. All the mathematical models were solved on the precise geometry of male and female *P. cieloi* AT, which were obtained through *μ*-CT scans and three-dimensional reconstruction (see §2.3). The sound propagation in the AT was first considered in the *frequency domain*, with the sound pressure satisfying the Helmholtz equation [24].

The AT wall was assumed to be rigid. To account for the viscous losses due to boundary layers formed near the wall, the Boundary Layer Impedance condition was applied at the wall [25,26]. A harmonic wave of magnitude 1 Pa was modelled as the incident wave entering the domain through a surface representing the spiracle. To reduce any reflections from the domain opening, the incident wave was also coupled with a Plane Wave Radiation condition at the spiracle [27].

The solution was also considered with a modal analysis. Using the *Eigenfrequency* study of COMSOL Multiphysics [23] for the system of equations described above, the first eigenfrequency and the corresponding quality factor (*Q* factor) were calculated by the following formula in-built in COMSOL,

2.1 Q=2|f1|Im(f1),

where *f*_1_ is the first eigenfrequency, and Im( · ) denotes the imaginary part of a complex number.

The variational form of the formed boundary-value problems were solved by the finite-element method, where quadratic Lagrange polynomials were employed for a third-order accuracy in the *L*_2_ norm [28]. The solution was considered in the frequency range 2–80 kHz, with a resolution of 100 Hz. The mesh size was taken so that there were at least ten tetrahedral elements per wavelength at the largest frequency of 80 kHz. A boundary layer mesh was also constructed near the AT wall to resolve the viscous boundary layers and it was connected to the tetrahedral mesh to form a conforming mesh. In total, four boundary layers composed of prisms were employed, with the thinnest boundary having a width of 4 μm. The layers had a stretching factor of 1.5.

In addition to the frequency domain solutions, the problem was also numerically solved in the time domain. In this case, we considered the solution to a wave equation accounting for dissipation due to viscous losses. This was achieved using the General dissipation equation in COMSOL Multiphysics, v. 5.6 [23], available in the *Pressure Acoustics, Transient* node. The initial condition was taken as zero for sound pressure everywhere in the domain and the Sound Hard Boundary condition was applied at the AT wall [24]. A Plane Wave Radiation condition was again applied at the spiracle, where a harmonic incident wave of frequency 11 kHz (*P. cieloi* calling song frequency) and magnitude 1 Pa was modelled to enter the AT. Using the solution obtained from the formed system of time domain equations, the speed at the distal end of the AT was calculated using the procedure described in [14].

For the numerical simulations in the time domain, the finite-element method was applied for the spatial variables, using the same mesh and polynomials as for the frequency domain solution. For the time variable, the generalized alpha method was applied, with a time step of Δ*t* = 1/(60 × 60 kHz) and total time *T* = 2.5 × 10^−4^ s. A time-step sensitivity analysis showed solution stability for the used Δ*t*.

### 2.5. LDV recordings of tympanal response

The experimental procedure was carried out as detailed in [1,8], for which we give a summary below. The experiments were conducted on a Melles Griot Optical Table Breadboard, Pneumatic Vibration Isolation (1 × 1 m area) (Melles Griot; Rochester, NY, USA), on which the LDV (Polytec PSV- 500-F; Waldbronn, Germany) was also placed. A loudspeaker (Ultrasonic Dynamic Speaker Vifa; Avisoft Bioacoustics) with a custom-designed plastic probe adapter was positioned 2 mm away from the spiracle ipsilateral to the bush-cricket ear. Due to the difficulty of flattening the spectrum, which was caused by the probe speaker, sound was delivered without amplitude correction for each frequency. Pressure was measured using a 1/8 inch precision pressure microphone (Brüel & Kjær, 4138; Nærum, Denmark) and a preamplifier (Brüel & Kjær, 2633), placed 2 mm away from the probe tip. A sound-level calibrator (Brüel & Kjær, 4231) was used to calibrate the microphone’s sensitivity.

The TM was acoustically stimulated only through the AT, with a probe loudspeaker. A platform that allows for the isolation and control of external and internal ear inputs was applied during this procedure (see [1]). A periodic chirp was used as the sound stimulus with a frequency interval ranging from 2 to 80 kHz. The overall duration of a full chirp was 32 ms, with a resolution of 1.95 μs (at a sampling rate of 512 kHz). A ramp was not used for the periodic chirps, which were generated from the Polytec software (PSV 9.2), passed to an amplifier (A-400, Pioneer, Kawasaki, Japan) and finally sent to the loudspeaker. The vibration displacement of the TM was measured using the LDV with a PSV-A-410 closeup unit. For analysing the mechanical response, recordings of the vibration displacement from the TM and the sound stimulus at the spiracle were taken at the same time. Placing the probe speaker 2 mm away from the spiracle allowed us to place the 1/8 inch microphone behind the tip of the probe, while leaving enough space for the stimulus to enter the spiracle. The LDV was operated to record vibrations using both the single-shot mode and the TM surface as a whole. The data collected was then used to calculate the displacement magnitude and frequency transfer functions of the tympanal response with input only through the AT path. Transfer functions were computed directly in the Polytec software in the frequency domain.

## 3. Results

Firstly, we carried out a spectral analysis of the recorded male and female calling songs. Taking both empirical and numerical approaches, we then conducted an analysis on the sound transmission properties of the dimorphic male and female *P. cieloi* AT. The tracheal morphological differences between the sexes were also analysed through the *μ*-CT images of the tubes.

For the results below presented in the form *a* ± *b*, *a* represents the mean and *b* is the standard deviation.

### 3.1. Calling song analysis

The calling songs of five female *P. cieloi* were recorded as outlined in §2.2. The calling songs of males are well known [22], hence we only recorded two male calling songs for easier comparison. Figure 1*d*,*f* demonstrates the calls and their spectral decomposition for a male (figure 1*d*) and a female (figure 1*f*) specimen. The frequency analysis of the recordings showed that for both of the sexes, the calling songs had a dominant frequency around 11 kHz (female 11.46 ± 2.7 and male 10.985 ± 0.12). As can also be observed from figure 1*d*,*f*, the male song had a much higher intensity compared with the female song. At the dominant frequency, the male song had a magnitude of 4.0 Pa (approx. 106 dB SPL), compared with the female song, which at the dominant frequency had a magnitude of 0.01 Pa (approx. 54 dB SPL). Furthermore, it was observed that the duration of the male calling song was significantly longer compared with the female song. While the male song lasted around 2 s, the female song had a duration of 0.02 s. The female song, however, was observed to have a wider frequency range than the male calling song (electronic supplementary material, figure S4), ranging up to 60 kHz with a significantly decreasing intensity as the frequency increased from 11 kHz. In particular, the relative intensity of the female song at higher frequencies was observed to be at least 10 dB lower than the dominant frequency.

For many Phaneropterinae species, the male produces two song elements: a complex one for species recognition and a simple one for triggering a fast female response [29,30]. The proposed triggering pulse in the call of male *P. cieloi* is shown in figure 1*d*. At 26.5°C, the latency time of the female (defined here as the interval between the beginning of the male triggering pulse and the beginning of the female response) to real male calls was 60.72 ± 6.78 ms (*n* = 12). We report here the latency of the female response and the triggering event of the male as general information; however, not being the main purpose of the paper, this topics deserves further attention.

### 3.2. Morphological measurements of the tracheae

**Figure 2.**
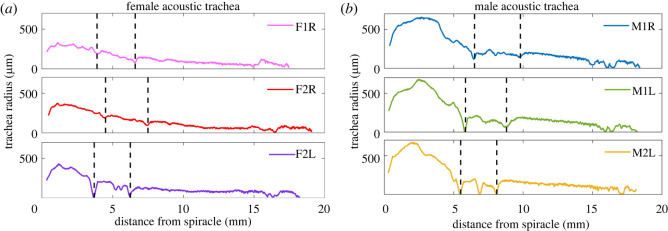
The morphological measurements of (*a*) three female and (*b*) three male *P. cieloi* acoustic trachea. The dashed black lines represent the location of the discontinuities along the trachea.

The measurements of three male and three female *P. cieloi* AT were carried out using the protocol outlined in §2.3. Figure 2 demonstrates the cross-sectional area radius of the AT with respect to the distance from the spiracle. These measurements showed a dimorphism between the ATs of the female (figure 2*a*) and the male (figure 2*b*). Further, the male spiracle had a surface area of 0.2740 ± 0.0365 mm^2^, compared with the female spiracle surface area of 0.15 ± 0 mm^2^, showing that the males have a larger tracheal opening. The male AT volume was also measured to be larger, at 7.2180 ± 0.7979 mm^3^, compared with the female AT volume of 2.5867 ± 0.4350 mm^3^. However, the AT length was consistent between the sexes, at 17.89 ± 1.1379 mm for males and 18.26 ± 0.81 mm for females.

**Figure 3.**
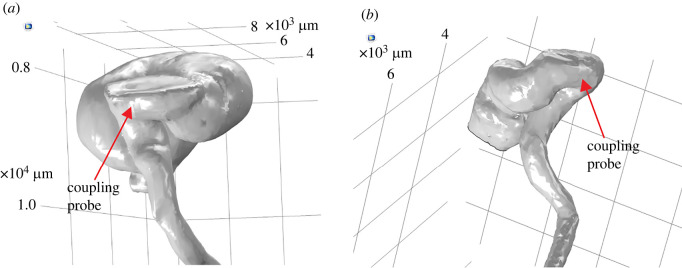
The ‘coupling probe’ between the bulla and the acoustic trachea for (*a*) a male and (*b*) a female specimen.

The morphological measurements also demonstrated that the AT of *P. cieloi* are not smooth tubes, since there were discontinuities at two distinct places along the measurement curves for both sexes (figure 2). The first discontinuity can be observed at the end of the bullae and the second is after a distance of about 3 mm from this. These discontinuities suggest that the bulla is not smoothly attached to the remainder of the AT. Instead, the two parts are connected with a short tubular section. This is further confirmed from the three-dimensional reconstruction of the AT, where figure 3 demonstrates this short tubular section acting as a ‘coupling probe’ between the bulla and the rest of the AT. It can also be observed from figure 2 that after the second discontinuity, the AT has a length of approximately 12 mm, with a linearly decreasing radius from 130 to 40 μm for females, and from 170 to 70 μm for males. Hence, the second part of the AT has the properties of a conical tube.

**Figure 4.**
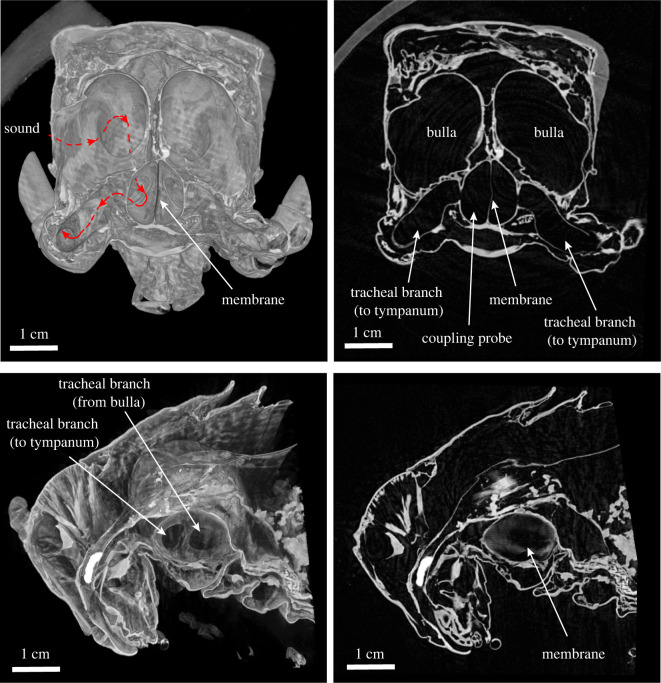
The *μ*-CT images of the membrane (septum) below the bulla, connecting the left and right trachea of a male *P. cieloi*. The red arrows show the route of the sound stimulus as it travels through the acoustic trachea.

The dissection of the male and female *P. cieloi* trachea also revealed a connection between the left and right ATs of these species (see figure 4; electronic supplementary material, figure S3). It was observed that the female ATs were only joined by a thin filament (electronic supplementary material, figure S3*a*), whereas the male ATs were strongly joined below the bulla (electronic supplementary material, figure S3*b*). Further investigations through *μ*-CT images of the male *P. cieloi* ATs have revealed a septum shared by the left and right ATs (figure 4).

### 3.3. Numerical calculation of tracheal sound transmission properties

The propagation of sound was simulated in the precise geometry of the dimorphic male and female ATs (see §§2.3 and 2.4). The incident wave was taken as a harmonic wave with a magnitude of 1 Pa, and the calculations were carried out in both the frequency domain and the time domain. The numerical results were obtained using three male and three female ATs.

**Figure 5.**
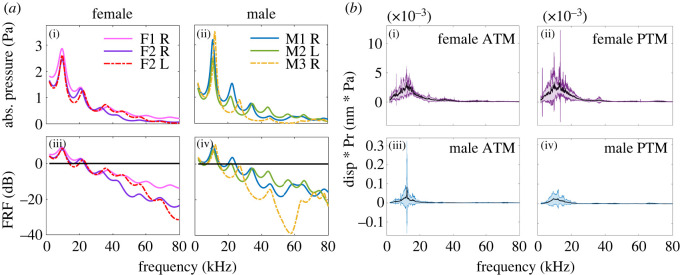
Numerical and experimental results at the tympana. (*a*) Numerical results recorded at the distal end of the *P. cieloi* acoustic trachea (AT): Absolute pressure obtained at the distal end of (i) three female and (ii) three male ATs; frequency response function (FRF) results at the distal end of (iii) three female and (iv) three male ATs, the black line is located at zero gain/loss. (*b*) The displacement magnitude of *P. cieloi* anterior tympanic membrane (ATM) and posterior tympanic membrane (PTM): displacement magnitude recorded at the (i) female ATM (*n* = 9), (ii) female PTM (*n* = 12), (iii) male ATM (*n* = 11) and (iv) male PTM (*n* = 13). The black curves denote the mean and the shaded regions denote the standard deviation.

Figure 5*a* presents the absolute sound pressure and the frequency response function (FRF) results at the distal end of the tube. The FRF calculations were done using the formula

3.1 $$H(f)=\frac{Y(f)}{X(f)},$$

where *f* is frequency, *H* is the frequency response, and *X*, *Y* are the input to and output from the system in the frequency domain, respectively, in Pa. The obtained results were then converted to dB.

**Figure 6.**
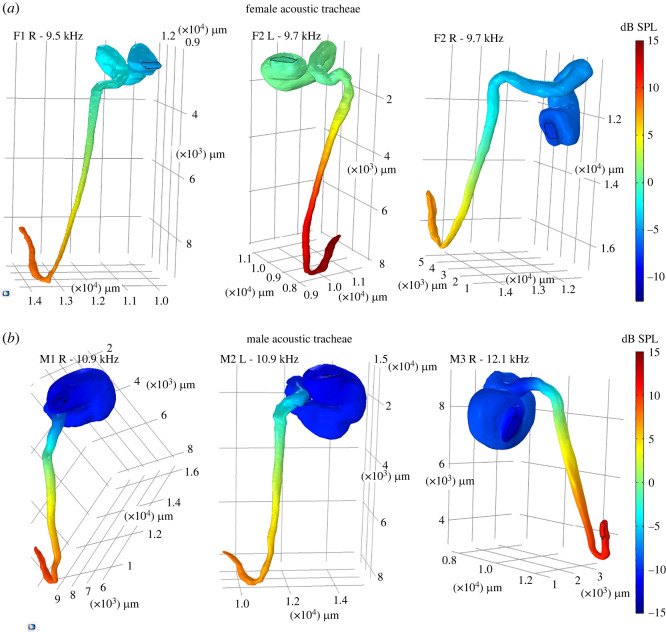
The magnitude of pressure difference, with respect to the incident wave magnitude, inside the *P. cieloi* acoustic trachea (AT). The numerical simulation of the pressure difference magnitude in (*a*) three female and (*b*) three male ATs, at their individual resonant frequencies.

As can be observed from figure 5*a*, the numerical results show that the *P. cieloi* AT has a resonant frequency of approximately 11 kHz (male = 11.3 ± 0.693 kHz, female = 9.63 ± 0.116 kHz). The pressure gain at the resonant frequencies is calculated as 8.426 ± 0.664 dB for females and 9.597 ± 1.527 dB for males. The difference between the calculated sound pressure magnitude inside the AT and the incident wave magnitude is also given in figure 6*a* for females and figure 6*b* for males, at the resonant frequency of each trachea. As can be seen from these figures, the sound pressure magnitude is heavily dampened in the bulla. After passing through the bulla, the sound pressure shows a gradual increase in magnitude from the proximal to the distal end, reaching its maximum at the tympana for both of the sexes.

In addition to a numerical frequency analysis, a modal analysis was also carried out to determine the fundamental frequency of the AT (see §2.4 for details of the applied procedure). The results showed the male AT had a fundamental frequency at 12.026 ± 0.3379 kHz, with a *Q* factor of 21.167 ± 8.9. Similarly, the results from female AT showed a fundamental frequency of 11.0237 ± 0.1175 with a corresponding *Q* factor of 17.2864 ± 4.383.

Finally, using the time domain results, the sound speed was calculated at the distal end of the AT for a harmonic incident wave of 1 Pa and 11 kHz. For females, the sound speed was obtained as 190.5863 ± 21.5379 m s^−1^, with a mean 44.5% lower than the speed of sound in air (343 m s^−1^), whereas for males the sound speed was 222.6495 ± 39.3671, with a mean 35% lower than the speed of sound in air.

### 3.4. Tympanal response to sound pressure

The sound transmission properties of male and female *P. cieloi* ATs were investigated experimentally by recording the tympanal response to sound. The recordings were obtained using an LDV, as outlined in §2.5. Figure 5*b* demonstrates the cross-power spectrum of the tympanal displacements and the incident wave in the frequency range 2–80 kHz, where the incident wave had a uniform amplitude for all the experiments.

From figure 5*b,* it can be observed that the maximum tympanal displacements for both male and female specimens are around 11 kHz: female ATM 12.46 ± 2.96 kHz (*n* = 9), PTM 12.30 ± 3.86 kHz (*n* = 12); male ATM 11.5 ± 1.88 (*n* = 11), PTM 12.04 ± 2.16 (*n* = 13). In the interval 20–80 kHz, tympanal displacements were not significant for either of the sexes. The displacement magnitude, however, showed a variation between the sexes, with a significantly higher magnitude for males compared with females. However, a large standard deviation was obtained at the maximum displacement for both the sexes: female ATM 0.0034 ± 0.0028 nm · Pa (*n* = 9), PTM 0.0044 ± 0.0079 nm · Pa (*n* = 12); male ATM 0.0829 ± 0.1553 nm · Pa (*n* = 11), PTM 0.0263 ± 0.0307 nm · Pa (*n* = 13).

## 4. Discussion

In this study, we took an interdisciplinary approach to investigating the underlying mechanism for the transmission of sound in the AT of the bush-cricket *P. cieloi*. To this end, we obtained the LDV recordings of the tympanal response to the sound stimulus through the AT. Furthermore, to analyse the effect of the tracheal design geometry on the sound transmission properties, we also obtained the precise morphological measurements of the dimorphic male and female ATs from *μ*-CT images. After three-dimensional reconstruction, *μ*-CT images were also used for the FEA of sound propagation in the AT of both sexes. Finally, a spectral analysis of the male and female calling songs was carried out to examine any parallels between their spectral ranges and the AT transmission properties. While the male calling song was also investigated by Buzetti *et al.* [22], in this study we analysed the properties of the female calling song for the first time.

One of the most commonly observed tracheal designs in bush-crickets is the exponential horn-shaped ATs, which are smooth tubes whose radius-length data have an exponential fit (see [1,5,6,11,12]). These tracheal tubes have been observed to act as high-pass band filters, producing a pressure gain up to 20 dB above a certain cut-off frequency [31,32]. For these species, the mating call frequency has been observed to be above the cut-off frequency [11], significantly improving the hearing ability of the bush-cricket for picking up conspecific calls. Moreover, it has been noted that these ATs lead to a reduction of the sound speed by up to 25% compared with the free-field sound speed in air [1,14].

Our measurements of the *P. cieloi* AT, however, have revealed morphological qualities different than an exponential horn design (see figure 2; electronic supplementary material, figure S5). For instance, the *P. cieloi* trachea is not smooth. The bulla, which forms a closed chamber, is attached to the rest of the trachea through a short tube that acts as a coupling probe. Our analysis also revealed a dimorphism between the male and female ATs, which showed the male spiracle and bulla to be considerably larger compared with the female. The remaining, tubular part of the AT was observed to have a conical shape with a length of 12 mm, where the male had a radius 30–40 μm larger than the female. Hence, our measurements suggest that the AT is formed of two distinct parts (a closed chamber—the bulla—and a conical tube) coupled through a probe. As a result, the influence of both parts of the AT need to be accounted for to understand their combined effect on the sound pressure observed at the distal end of the tube.

The different tracheal design of *P. cieloi* from an exponential horn also resulted in differing acoustic properties (see electronic supplementary material, figure S6 for a comparison of pressure gain differences resulting from the discrepancies in tracheal morphologies). Rather than an increased pressure magnitude after a cut-off frequency, both numerical (figure 5*a*) and experimental (figure 5*b*) results suggested that the male and female *P. cieloi* ATs have a resonant frequency around 11 kHz, which is the dominant frequency of the calling songs for both the sexes (figure 1*d*,*f*). While the female calling song showed a wider frequency spectrum (electronic supplementary material, figure S4), the relative intensity was significantly lower at higher frequencies compared with the dominant frequency. Hence, the resonant frequency of the ATs allowed the male *P. cieloi* to concentrate their hearing abilities on the most relevant frequency of the female calling song. Numerical calculations have also shown the AT to have a large *Q* factor at this frequency (21.167 ± 8.9 for males and 17.2864 ± 4.383 for females), so that a pressure gain is observed in the range 2–20 kHz at the distal end of the tube. Hence, the *P. cieloi* ATs function as sharply tuned band-pass filters rather than as exponential horns. It is generally claimed that the resonant frequency of this type of AT is dependent on the length of the trachea [4,13]. However, in these studies, the transmission properties of the tube were analysed based on the pressure recordings at the distal end of the AT, or the tympanal response due to LDV recordings. Hence, the pressure distribution inside the tube was not investigated. In this study, we have also numerically investigated the sound pressure distribution inside the *P. cieloi* AT.

As can be observed from figure 6, the numerical results show that at the resonant frequencies, the bulla does not contribute to the pressure gain. Instead, it has a dampening effect on the sound as it enters the tube. A similar attenuation of sound was also observed throughout the frequency range 2–80 kHz (see electronic supplementary material, figures S1 and S2, for 20, 40 and 60 kHz). However, the increasing pressure magnitude in the tubular part of the AT suggests that it acts as an open–closed conical air column. Such a pipe will have the same natural frequencies as an open–open cylindrical pipe [33]. It is estimated that for the conical part of the *P. cieloi* AT, the slant length is about 13–14 mm. For an ideal conical tube of this slant length, the fundamental frequency can be easily calculated as 12.3 kHz, which is in accordance with the AT fundamental frequency. This also elucidates the similar transmission qualities observed between the sexes, despite major differences in the sizes of their spiracle and bulla. It is worth noting, however, that the damping in a male bulla is higher than in the female bulla in the range 2–80 kHz (see figure 6 for the resonant frequencies). Contrarily, the pressure gain in the male conical horn is higher compared with the female. While the natural frequencies of a conical tube depend on the slant length rather than the radius, the radius is generally associated with the magnitude of the obtained pressure gain. Hence, the small discrepancies in the AT conical part radii of the two sexes appear to provide the required geometrical difference to obtain a similar pressure gain at the tympana, and to overcome the dissipation caused by the bulla at the resonant frequency.

A conical pipe, also referred to as a conical horn, does not have a cut-off frequency such as the exponential horn. The frequencies producing resonance are very close to the tube’s natural frequencies, although the resonance peaks broaden, and the minima between the peaks increase with increasing frequency [34]. This property cannot be observed in the pressure gain at the end of the AT, providing further evidence that the bulla has an increasing damping effect on the sound stimulus with the increase of frequency (figure 5*a*). Hence, the function of the bulla is to act as a cavity resonator for the purpose of sound attenuation rather than enhancement [35,36]. As a result, the AT should be considered as two resonators (the bulla and conical tube) coupled through a probe. Even though the effect of such couplings is a topic of ongoing research, it has been shown that it would have an effect on the resonant frequency and the transmission effects of the system [37]. Hence, the small difference observed between the resonant frequency of an idealized cone (12.3 kHz) and the *P. cieloi* AT (11 kHz) can also be attributed to this complex connection.

In terms of the theories related to the workings of the ‘resonator’ AT, our results suggest a more complex mechanism behind this process than just having a resonant frequency dependent on the length of the tube. The bulla plays a major role in attenuating the sound as it enters the tube, preventing any pressure gain at the natural frequencies of the conical tube other than around the fundamental frequency. This enables the bush-cricket to be more focused on the calling song frequency. Thus, the bulla prevents the conical horn part of the AT producing unnecessary pressure gains. This suggests that the underlying mechanism behind the AT working as a cavity resonator is due to a combined effect of the two coupled resonators, which is the main result of this study.

The bush-cricket *P. cieloi* belongs to the subfamily Phaneropterinae, which often displays duetting during courting; an uncommon strategy for acoustic communication in bush-crickets. The male will produce a calling song to attract a potential mate and will wait for the female to reply acoustically. The answer will come from the female in the form of another conspecific call. Even though both male and female *P. cieloi* calls have the same dominant frequency of approximately 11 kHz (figure 1*d*,*f*), the female calling song is much shorter in duration and is considerably lower in intensity, giving the male a very small window for hearing and localizing. The short female answer to the longer male courting song is a characteristic of many Phaneropterinae species [38–40]. As expected, the experimental and numerical results uniformly showed that despite morphological differences, both male and female *P. cieloi* ATs produced the highest pressure gain around 11 kHz (figure 5*a*,*b*). These results support our hypothesis that both male and female ATs are tuned to the frequency corresponding to the shared mating call frequency. Nevertheless, some qualitative differences of sound transmission were also observed between the male and the female ATs. For instance, the *Q* factors at the resonant frequency had some differences between the sexes (21.167 ± 8.9 for males and 17.2864 ± 4.383 for females), suggesting a higher sharpness of tuning in the male ear compared with the female ear. This can also be observed in figure 5. This quality can be considered as a trait of the male ear to help distinguish the low intensity female song from any background noise. The same weak correlation as with male quality may be found in the species recognition function of the song, which does not apply to the female song.

While the numerical results did not show any significant differences in pressure gain magnitude between the sexes (maximum pressure gain 9.597 ± 1.527 dB for males and 8.426 ± 0.664 dB for females), the experimental results demonstrated a higher tympanal response for males compared with females (female ATM 0.0034 ± 0.0028 nm · Pa, PTM 0.0044 ± 0.0079 nm · Pa; male ATM 0.0829 ± 0.1553 nm · Pa, PTM 0.0263 ± 0.0307 nm · Pa). This strongly suggests that the observed differences are not due to tracheal design, since the numerical simulations were carried out on the precise tracheal geometry. However, the mathematical models make the simplifying assumption that the AT wall is rigid, and they do not account for the tympana material properties. Hence, we conjecture that the discrepancies in gain are a result of potential differences in the TM material properties between the sexes. From the experimental results, it can be suggested that the male ears are more sensitive to sounds at 11 kHz as an adaptation to the very low intensity of the female calling song.

In the numerical simulations, we did not take into account the conjoined morphology of the male *P. cieloi* left and right ATs (figure 4) due to a lack of information of the septum material properties and the lack of experimental LDV data for cross-talk evidence, as the complex anatomy was discovered in preserved experimental voucher specimens. A difference in the material properties of the male AT at the septum could potentially account for the observed pressure gain discrepancies, as well as create the possibility of cross-talk between the male left and right ATs [41,42]. In field crickets, cross-talk has generally been associated with aiding the directional response to conspecific signals [41–45]. This cross-talk is facilitated by a median septum, which is a double membrane that occurs in the left and the right trachea medial joint, and does not contain tracheal taenidia. In other words, the septum is formed by two membranes of independent trachea [43]. This internal connection facilitates three potential sound inputs to the TM: two originating from the acoustic spiracles located on the thorax and a third from the contralateral TM [44]. Transmission from the contralateral spiracles involves an additional phase shift due to the medial septum [44–48] and this varies steeply with sound frequency [49]. In field crickets, ablation of the septum changes essential characteristics in the phonotactic behaviour [44] and reduces the amount of interaural intensity differences available for localization from 10 dB to about 2 dB [46]. For crickets, this mechanism is effective at around 5 kHz. Although we do not have experimental evidence that the duetting *P. cieloi* use cross-talk, we speculate that a cross-talk between the male trachea could provide additional acoustic inputs, allowing for an increased localization ability of the female calling song despite its short duration. While beyond the scope of this study, an experimental cross-talk analysis between the tracheae of male *P. cieloi* certainly merits further investigation. Indeed, biophysical models in future works should look to include experimentally derived properties for tracheal structures and cross-talk effects, if available, to ensure accurate conclusions from modelling.

The numerical calculations of the speed of sound in the AT suggest a very high reduction compared with the free-field speed of sound in air (44.5% reduction for females and 35% for males). It can be argued that such an enhanced reduction increases the ability of *P. cieloi* to detect the conspecific calls, as the bush-cricket ear is endowed with a dual-input system (external and internal) and the speed reduction causes large delays of the internal acoustic version, improving their sound localization abilities [16]. The phase angle (in °) of the sound wave at the tympana can be calculated using the formula

$$\varphi^{\circ }=360^{\circ }\times f\times \Delta t,$$

where φ is the phase angle, *f* is the frequency and Δ*t* is the time delay. It is easy to calculate that an approximate AT length of 19 mm and a reduced wave speed of 190 m s^−1^ would lead to a time delay of 0.045 ms for the sound to reach the TM. Hence, at 11 kHz we have φ° = 178.2°, which is almost a perfect antiphase to the sound stimulus at the spiracle. This suggests that at the calling song frequency, the *P. cieloi* AT amplifies the TM response with no phase difference between the spiracle and TM; a quality enabling the delivery of maximum intensity to the TM. While this result is based on numerical calculations, further investigation of this property through experimentation is certainly worth pursuing.

In species with extremely short female replies such as *P. cieloi*, it has also been observed that males may use the intermission between their calling song and the female reply as a measure to estimate the distance to the female [47], giving the species another technique for locating their potential mate. Nevertheless, in their natural habitat *P. cieloi* generally live in close proximity, with 8–10 individuals of both sexes coexisting on the same host plant. Hence, it might not be necessary for the male ears to use the female song latency of reply for estimating the distance. Another significance of the mating call quality between duetting bush-crickets was identified by Tuckerman *et al.* [50], where it was shown that male song duration and complexity were significant factors for the female when choosing a mate. For instance, for the species *Scudderia curvicauda*, the length of the male signal was observed to be proportional to body mass [50]. With the implication of the body mass on the size of the nuptial gift formed of a proteinaceous mass surrounding the sperm ampulla, the spermatophylax, the females were more inclined to seek out mates with longer mating calls. While forming predictions about the ecological implications of *P. cieloi* mating calls is beyond the scope of this study, the argument provided by Tuckerman *et al*. [50] provides another possible explanation for the longer duration of the male *P. cieloi* mating call compared with the female call.

In conclusion, this study elucidates a novel mechanism for investigating auditory sensitivity in bush-crickets, offering insights into the diverse functions of the AT between the species. Based on the results presented, it is presumed that future works examining AT function across other bush-cricket taxa will likely uncover multiple innovative solutions to the problems they face in hearing. Hence, as a continuation of this work, a comprehensive study examining AT function across other bush-cricket taxa is certainly worth pursuing.

## Data Availability

The constructed STL files for the trachea geometries used in the numerical models (for six specimens) and the set-up of the three different numerical models (developed in COMSOL Multiphysics, v. 5.6) are available from the Dryad Digital Repository: https://doi.org/10.5061/dryad.n2z34tn0j [51]. The data are provided in electronic supplementary material [52].
